# The HDAC Inhibitor FK228 Enhances Adenoviral Transgene Expression by a Transduction-Independent Mechanism but Does Not Increase Adenovirus Replication

**DOI:** 10.1371/journal.pone.0014700

**Published:** 2011-02-17

**Authors:** Angelika Danielsson, Helena Dzojic, Victoria Rashkova, Wing-Shing Cheng, Magnus Essand

**Affiliations:** Department of Immunology, Genetics and Pathology, Science for Life Laboratory, Uppsala University, Uppsala, Sweden; Cleveland Clinic, United States of America

## Abstract

The histone deacetylase inhibitor FK228 has previously been shown to enhance adenoviral transgene expression when cells are pre-incubated with the drug. Upregulation of the coxsackie adenovirus receptor (CAR), leading to increased viral transduction, has been proposed as the main mechanism. In the present study, we found that the highest increase in transgene expression was achieved when non-toxic concentrations of FK228 were added immediately after transduction, demonstrating that the main effect by which FK228 enhances transgene expression is transduction-independent. FK228 had positive effects both on Ad5 and Ad5/f35 vectors with a variety of transgenes and promoters, indicating that FK228 works mainly by increasing transgene expression at the transcriptional level. In some cases, the effects were dramatic, as demonstrated by an increase in CD40L expression by FK228 from 0.3% to 62% when the murine prostate cancer cell line TRAMP-C2 was transduced with Ad[CD40L]. One unexpected finding was that FK228 decreased the transgene expression of an adenoviral vector with the prostate cell-specific PPT promoter in the human prostate adenocarcinoma cell lines LNCaP and PC-346C. This is probably a consequence of alteration of the adenocarcinoma cell lines towards a neuroendocrine differentiation after FK228 treatment. The observations in this study indicate that FK228 enhances adenoviral therapy by a transduction-independent mechanism. Furthermore, since histone deacetylase inhibitors may affect the differentiation of cells, it is important to keep in mind that the activity and specificity of tissue- and tumor-specific promoters may also be affected.

## Introduction

Viral vectors based on human adenovirus serotype 5 (Ad5) are the most commonly used vectors for gene therapy and they are frequently used for vaccination. In cancer gene therapy their effect can be limited due to that malignant cells often have low expression of the coxsackie adenovirus receptor (CAR), which is the primary receptor for Ad5 [1]. Different means to increase CAR expression on target cells would presumably lead to enhanced adenoviral gene therapy efficacy.

Regulation of gene expression is controlled by various mechanisms. Histone acetylation plays a key role in transcriptional control by modulating chromatin structure [2]. In general, histone acetylation is associated with relaxation of chromatin and activation of transcription whereas histone deacetylation is associated with condensation of the chromatin structure and repression of transcription. Histone deacetylase inhibitors (HDACi) cause accumulation of acetylated histones, which affect transcription of specific genes and both up- and down-regulation of gene expression can occur [3]. FK228, also known as depsipeptide or romidepsin, is an HDACi that can induce cell cycle arrest, promote apoptosis and inhibit angiogenesis [4]. FK228 triggers both mitochondrial-dependent [5] and mitochondrial-independent [6] apoptosis and is more toxic to malignant than non-malignant cells [7]. Because of its direct cytotoxic effects, FK228 has been and is currently being evaluated in clinical cancer trials [8], [9]. FK228 has minimal antitumor activity in patients with metastatic castration-resistant prostate cancer [10]. In combination with docetaxel however, FK228 is able to enhance the antitumor effect both *in vitro* and in prostate cancer xenograft mouse models [11], [12]. In addition, FK228 has been shown to enhance the effect of adenoviral-mediated therapy [13], [14]. The mechanisms for this enhancement are not fully understood but upregulation of CAR has been suggested as one possibility [7], [15], [16], [17], [18].

Herein, we investigate the effect that FK228 has on adenoviral transduction and transgene expression in cancer cells. Upregulation of CAR plays if any, only a minimal role in the enhancement of transgene expression. Instead, we show that FK228 has the highest effect when administered directly after transduction, implying that it has a direct effect on adenoviral transgene expression, probably by a general increment in transcription of the host cell.

## Materials and Methods

### Cell lines

The prostate adenocarcinoma LNCaP, the colon carcinoma HT-29 and the glioma U343 were cultured in RPMI-1640 supplemented with 10% fetal bovine serum (FBS), 10 mM HEPES and 1 mM sodium pyruvate. The prostate adenocarcinoma PC-346C was cultured in DMEM:F-12 supplemented with 2% FBS, 0.01% bovine serum albumin, 10 µg/ml epidermal growth factor (Sigma Aldrich, St. Louis, MO), 1% insulin-transferrin-selenium, 0.1 nM R1881 (NEN Life Science Products Inc, Boston, MA), 1.4 µM hydrocortisone (Sigma Aldrich), 0.6 ng/ml triiodothyronine (Sigma Aldrich), 0.1 mM phosphoetanolamine, 50 ng/ml cholera toxin (Sigma Aldrich), 0.1 µg/ml fibronectin (Sigma Aldrich) and 20 µg/ml fetuin (Sigma Aldrich). The foreskin fibroblast 1064SK and the human embryonic kidney 293FT were cultured in DMEM supplemented with 10% FBS and 1 mM sodium pyruvate. The endocrine pancreatic tumor cell line BON was cultured in DMEM with Glutamax-I and F12 K Nutrient Mixture (Kaighn's Modification) at a 1∶1 ratio, supplemented with 10% FBS and 1 mM sodium pyruvate. The murine prostate adenocarcinoma TRAMP-C2 was cultured in DMEM supplemented with 5% FBS, 5% NuSerum (Becton Dickinson, Franklin Lakes, NJ), 1 nM dihydrotestosterone (Sigma Aldrich) and 5 µg/ml insulin (Sigma Aldrich). All medium contained 1% penicillin-streptomycin. The cell culture reagents were from Invitrogen (Carlsbad, CA) except when stated otherwise. All cell lines were purchased from ATCC (Manassas, VA) except for BON that was a kind gift from Dr. CM Townsend (Galveston, TX) and TRAMP-C2 that was a kind gift from Dr. NM Greenberg (Baylor College of Medicine, Houston, TX).

### Isolation of peripheral blood mononuclear cells

Buffy coats from approximately 420 ml of blood were obtained from five healthy volunteers. Peripheral blood mononuclear cells (PBMC) were isolated by density centrifugation over Ficoll-Paque (GE Healthcare, Bucks, UK). The PBMC fraction was obtained, washed twice with PBS and resuspended in culture medium. The cells were divided into five T-75 cell culture flasks and separated by adhesion for 90 min. The non-adherent fraction (mainly lymphocytes) was removed, and the adhesion fraction (mainly monocytes) was washed twice with PBS, trypsinized, counted and used for transduction.

### Viral vectors

The recombinant serotype 5-based adenoviral vectors used in this study have been described previously: Ad[Mock] [19], Ad5 wt [20], Ad[i/PPT-LUC] [20], Ad[CMV-LUC] [21], Ad[i/PPT-E1A, E3] [20], Ad[CMV-GFP] [21], Ad[CD40L] [22], and Ad[CgA-LUC] [23]. One new vector, Ad[I/PPT-GFP] was made by replacing the luciferase transgene in Ad[I/PPT-LUC] [21] with the sequence encoding enhanced green fluorescent protein (GFP) by using HindIII and XbaI. A serotype 5 adenoviral vector with the fiber shaft and knob from serotype 35, Ad5/f35[CMV-GFP], was a kind gift from Dr Fan (Lund University, Lund, Sweden) and has been described and evaluated before [24], [25]. The vectors were produced, purified and titrated as described earlier [20].

A vesicular stomatitis virus (VSV)-G protein pseudotyped self-inactivating (SIN) lentiviral vector with a CMV-GFP expression cassette was produced, LN[CMV-GFP]. For lentiviral vector production, 293FT cells were grown in 15 cm plates to a confluence of 80–90% and transfected with 8.1 µg pRRL-CMV-GFP (kind gift from Dr. F Carlotti, Leiden University Medical Center, Leiden, The Netherlands), 4.05 µg pLP1 (gag/pol), 4.05 µg pLP2 (rev), and 4.05 µg pLP/VSVG using 60 µl Lipofectamine 2000 per plate. Viral supernatants were collected after 48 h, filtered (0.45 µm) and concentrated by ultracentrifugation at 75,000 g for 90 min at 4°C using a Sorvall AH629 rotor. The viral pellet was resuspended in PBS and stored at −80°C until further use. Viral titer was determined by the HIV-1 p24 Antigen ELISA from ZeptoMetrix Corporation (Buffalo, NY) according to instructions from the manufacturer.

### Cell viability assay

Prostate cancer cells were treated with various concentrations of FK228 (Fujisawa Pharmaceuticals Co., Ltd., Osaka, Japan) ranging from 0.1 ng/ml to 300 ng/ml and then plated in 96 well plates (7500 cells/well). After 24, 48 and 72 h, cell viability was determined by the MTS Cell Titer 96 Aqueous One Solution Proliferation Assay Kit (Promega, Madison, WI) according to the manufacturer's instructions. Average enzymatic activity from triplicate samples was expressed in relation to average activity in untreated cells.

### Green fluorescent protein reporter gene analysis

Cells were transduced in suspension for 2 h with Ad[CMV-GFP], Ad5/f35[CMV-GFP] and Ad[I/PPT-GFP] at various MOIs ranging between 0.1 and 300 FFU/cell depending on the cell line. Cells were also transduced with LN[CMV-GFP] using 0.3 vp/cell for all cell lines. Cells were either pre-treated with FK228 (3 ng/ml) for 24 h before transduction and/or incubated with FK228 2 h after transduction. Transduced monocytes were incubated with 1 ng/ml depsi. The HDACi valproic acid (VPA) from Sigma Aldrich was always added 2 h after transduction at a concentration of 5 mM. Cells were harvested 48 h after transduction and analyzed with FACSCalibur Flow Cytometer (San Diego, CA).

### Analysis of cell surface receptors

Cells were incubated with standard medium or medium containing FK228 (3 ng/ml) for 48 h at 37°C. Cells were then harvested and stained with antibodies (30 min, 4°C) for the following surface receptors using mouse monoclonal antibodies: CAR (RmcB hybridoma, ATCC, Manassas, VA), CD46 (BD Biosciences), αVβ3 (Chemicon, Temecula, CA) and αVβ5 (Chemicon). After washing, the cells were incubated with a FITC-labeled rabbit-anti-mouse antibody (Dako, Glostrup, Denmark) for 30 min at 4°C followed by washing. A mouse isotype-matched antibody (Dako) and the FITC-labeled secondary antibody were used as controls. The samples were analyzed by FACSCalibur Flow Cytometer. Mean fluorescence intensity (MFI) of receptor expression of FK228 treated cells was related to untreated cells.

### Analysis of CD40L expression

Cells were transduced with Ad[CD40L] for 2 h, washed and then incubated with medium containing various concentrations of FK228. Cells were stained 24 h thereafter with a PE-conjugated antibody against CD40L (Pharmingen, San Diego, CA). An isotype-matched PE-conjugated antibody (Pharmingen) was used as negative control. Cells were stained with antibodies for 15 min at room temperature. Expression of surface CD40L was analyzed by flow cytometry (FACSCalibur).

### Luciferase reporter gene assay

The luciferase reporter gene assay has been described earlier [20]. Briefly, LNCaP cells were transduced with Ad[i/PPT-LUC], Ad[CgA-LUC] and Ad[CMV-LUC] at an MOI of 50 FFU/cell and BON cells were transduced with Ad[CgA-LUC] and Ad[CMV-LUC] at an MOI of 10 FFU/cell in suspension for 2 h. Thereafter, fresh medium alone or fresh medium with FK228 (3 ng/ml) or VPA (5 mM) was added. After 48 h of incubation at 37°C cells were lysed and luciferase activity was measured using a Wallac VICTOR^2^ multilabel counter (PerkinElmer, Turku, Finland). Background levels were subtracted and luciferase activities were related to total protein concentrations in the samples (relative luciferase units per µg protein, RLU/µg).

### Replication assay

LNCaP and PC-346C cells were transduced with Ad5[i/PPT-E1A, E3] and Ad5 wt in suspension at an MOI of 10 and 1 FFU/cell respectively. BON was transduced with Ad[CgA-E1A] and Ad5 wt at an MOI of 1 FFU/cell. After 2 h the cells were washed and resuspended in medium with or without FK228 (3 ng/ml) or VPA (5 mM). After 72 h of incubation at 37°C, cells and medium were collected and viral DNA was purified using High Pure Viral Nucleic Acid Kit (Roche, Mannheim, Germany), according to the manufacturer's instructions. Viral DNA content was analyzed by quantitative real time PCR (qPCR) using the iCycler IQ real-time detection system (Bio-Rad, Hercules, CA). The specific PCR product was continuously measured during 40 cycles (95°C for 15 sec, 60°C for 60 sec) using iQ SYBR Green supermix (Bio-Rad) and primers in the adenovirus E4 region: 5'-CAT CAG GTT GAT TCA CAT CGG-3' (E4.Forward) and 5'-GAA GCG CTG TAT GTT GTT CTG-3' (E4.Reverse). All samples were amplified in triplicates. Copy numbers were related to a standard curve made by serial dilutions of a plasmid containing E4 open reading frame 1 (pCR2.1(Ad5E4orf1)). Relative replication was defined as the increase in copy number compared to input virus (also measured by qPCR).

### Gene expression

LNCaP and PC-346C cells were transduced with Ad[i/PPT-LUC] at MOI 10 FFU/cell and then treated with 3 ng/ml FK228 or 5 mM VPA for 48 h. Cells were then collected and RNA was extracted using RNeasy Mini Kit (Qiagen, Hilden, Germany). Three µg of total RNA and 1 µl SuperScript II Reverse Transcriptase (Invitrogen) were used for cDNA synthesis. The expression of prostate epithelial cell-associated genes [androgen receptor (AR), androgen receptor coregulator (ARA24), prostate-specific antigen (PSA), prostate-specific membrane antigen (PSMA), 5-α reductase type 1 and 2 (SRD5A1 and SRD5A2) and T cell receptor γ-chain alternate reading frame protein (TARP)] and neuroendocrine-associated genes [β-tubulin III (β-tubIII), chromogranin A (CgA), neuron-specific enolase (NSE) and synaptophysin (SYP)] was analyzed by quantitative PCR with the same protocol as for the replication assay, described above. The primer sequences are listed in Table S1. RNA levels in FK228 and VPA treated cells were related to RNA levels in untreated cells.

### Statistical analysis

Statistical analysis was done using the GraphPad Prism version 4.03 (GraphPad Software, San Diego, CA). One-way analysis of variance followed by Dunnet's post test was used to compare luciferase activities and replication. The p value is less than 0.05 for the luciferase assay and less than 0.0001 for the replication assay.

## Results

### FK228 above 3 ng/ml reduces viability of prostate cancer cell lines

**Figure 1:**
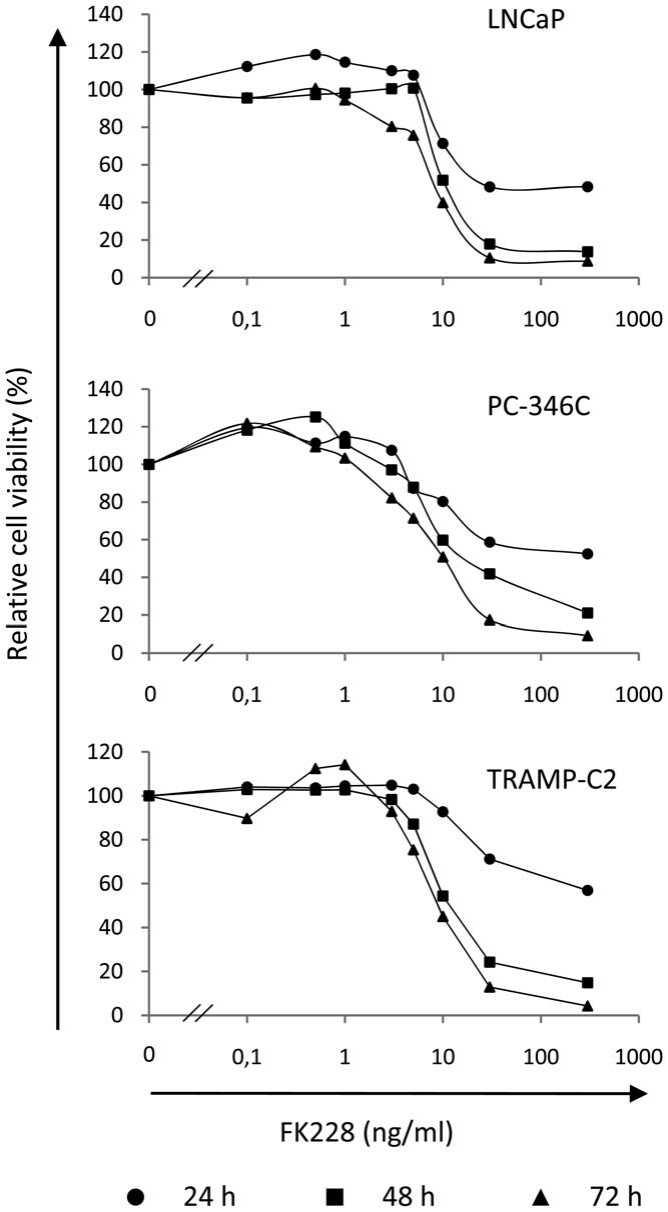
FK228 concentrations above 3 ng/ml reduce cell viability in prostate cancer cells. TRAMP-C2, LNCaP and PC-346C cells were treated with various concentrations of FK228 (ranging from 0.1 ng/ml to 300 ng/ml) for 24, 48 and 72 h. Cell viability measured by the MTS assay is expressed in relation to the activity in untreated cells. FK228 concentrations higher than 3 ng/ml reduced viability in all cell lines.

Cell viability studies were performed to determine FK228 toxicity for the human prostate cancer cell lines LNCaP and PC-346C and the mouse prostate cancer cell line TRAMP-C2. Cells were cultured in medium containing FK228 for 24, 48 and 72 hours before MTS assay analysis. After 72 h of incubation at 3 ng/ml FK228, around 80% of LNCaP and PC-346C cells were viable. TRAMP-C2 appeared to be somewhat less sensitive to FK228 than LNCaP and PC-346C. Reduced cell viability was observed at FK228 concentrations of 3 ng/ml and above for all cell lines and time points (Figure 1).

### Addition of FK228 after transduction enhances transgene expression of Ad[CMV-GFP] more than addition of FK228 before transduction

**Figure 2:**
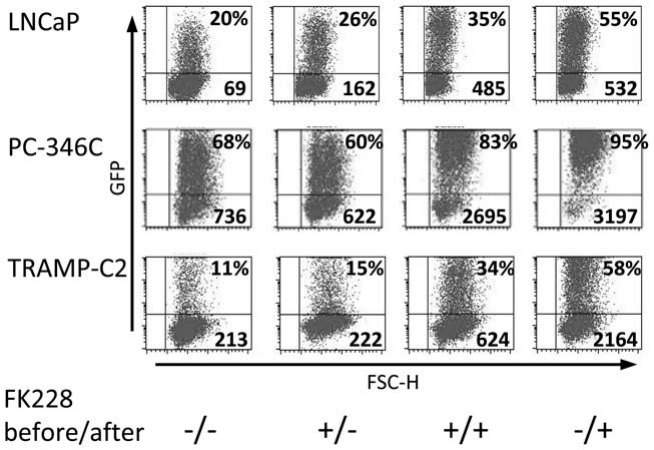
Addition of FK228 after adenoviral transduction enhances transgene expression. Prostate cancer cell lines were grown in medium with or without 3 ng/ml FK228 for 24 h (before). Cells were then transduced with Ad[CMV-GFP] for 2 h, washed and then cultured in medium with or without FK228 for another 48 h (after). The percentages of GFP positive cells (upper right) and MFI values (lower right) are given. The highest transgene expression was observed when FK228 was administered after transduction. One representative set out of three experiments is shown.

**Figure 3:**
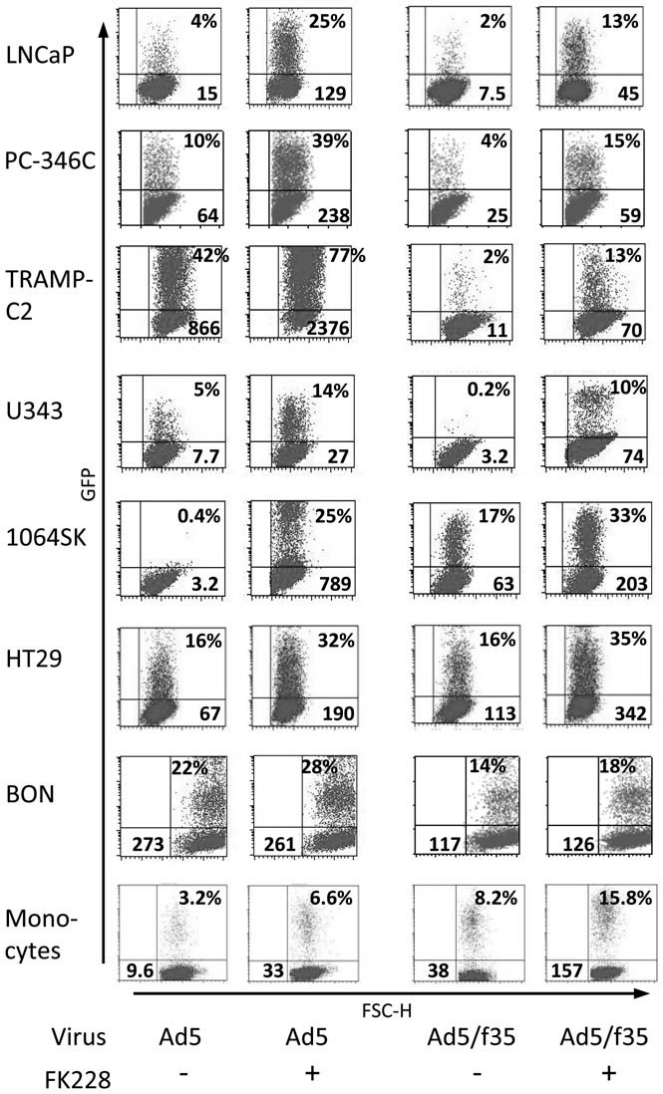
FK228 enhances transgene expression in various cell types. Cells were transduced with Ad[CMV-GFP] or Ad5/f35[CMV-GFP] for 2 h and then incubated for 48 h in medium containing 3 ng/ml FK228. The percentages of GFP positive cells (upper right) are given as well as MFI values (lower right or left). FK228 enhanced transgene expression of Ad5 virus and Ad5 virus with fiber shaft and knob from serotype 35 in all cell lines. Each cell line was evaluated three times with similar results. Monocytes from five different donors were evaluated. Representative examples are displayed in the figure.

We next wanted to investigate the effect of FK228 on adenoviral transduction. LNCaP, PC-346C and TRAMP-C2 were grown in medium with or without 3 ng/ml FK228 for 24 h. Subsequently, cells were transduced with Ad[CMV-GFP] for 2 h and then cultured in medium with or without FK228 for another 48 h. GFP expression was analyzed by flow cytometry. Pre-incubation of cells with FK228 enhanced GFP expression to some extent in LNCaP and TRAMP-C2 (Figure 2). Pre- and post-incubation gave an even higher level of GFP expression in all three cell lines. However, the highest increase was observed when FK228 was added after transduction, indicating a post-transductional effect of FK228. The fact that FK228 had the largest effect when given after transduction, both when looking at percentage of GFP positive cells and mean fluorescence intensity (MFI), indicates that the receptors involved in adenoviral transduction may play a less important role for enhanced transgene expression as previously proposed. Next, we used both the serotype 5-based vector Ad[CMV-GFP] and the adenoviral serotype 5 vector with fiber shaft and knob from serotype 35 Ad5/f35[CMV-GFP] to screen cell lines of various origin as well as freshly isolated human monocytes. Treatment of cells with FK228 after transduction enhanced GFP expression (both percentage and MFI) in all cell lines and monocytes for both viruses (Figure 3). The effect was not as pronounced for a lentiviral vector with regard to GFP positive cells as FK228 increased GFP expression of LN[CMV-GFP] in TRAMP-C2 cells but had no effect in the other cell lines tested (Figure S1). The MFI values on the other hand were increased in all cells except for BON and monocytes.

### FK228 does not upregulate cell surface receptors needed for adenoviral infection

To investigate if FK228 upregulate cell surface receptors needed for adenoviral infection (CAR and αVβ3 and αVβ5 integrins for Ad5 and CD46 for Ad5/f35), cells were treated with FK228 for 48 h and then stained for receptor expression and evaluated by flow cytometry. The receptor expression of FK228-treated cells was related to the expression in untreated cells (set to 1.00). Most cell lines did not show any marked difference in surface receptor expression after treatment of FK228 (values at 1.00±25%, Table S2). In the 1064SK fibroblasts the mean fluorescence intensity doubled for CAR and increased by 50% for αVβ3 after treatment with FK228. Some increase of CAR was observed also in U343 and the mean fluorescence value for αVβ5 in LNCaP and CD46 in PC-346C was reduced by approximately 50% upon treatment with FK228. Overall, the changes in receptor expression was if anything only marginal after FK228 treatment, further indicating that receptor upregulation is not the main reason for increased transgene expression after adenoviral transduction.

### FK228 treatment enhances adenoviral-mediated CD40L expression in prostate cancer cells

**Figure 4:**
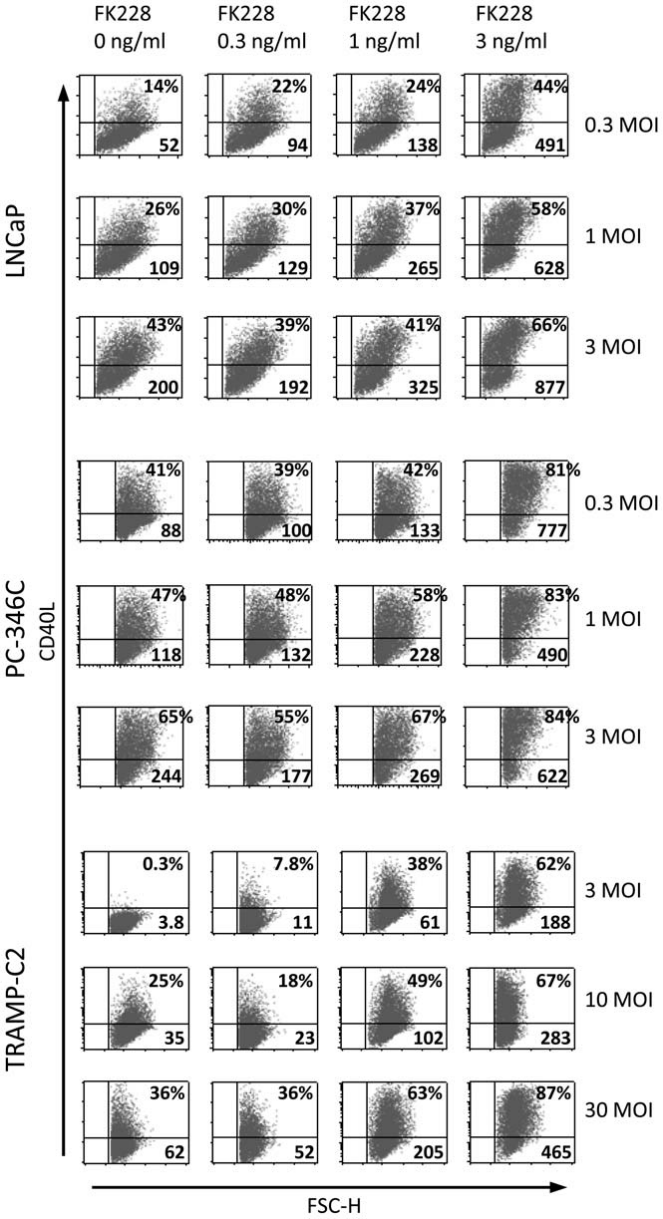
FK228 treatment enhances adenoviral-mediated CD40L expression. Prostate cancer cell lines were transduced with various MOI of Ad[CD40L] and cultured in medium with various concentrations of FK228 for 24 h. Cell surface expression of CD40L was analyzed by flow cytometry. Percentages of CD40L positive cells (upper right) and MFI values (lower right) are given. FK228 increased CD40L expression in a dose-dependent manner. One representative experiment out of three is shown.

Since FK228 had an effect on the GFP transgene we next wanted to investigate if it can increase also therapeutic transgene expression. An Ad5-based vector with the therapeutic CD40 ligand transgene driven by the CMV promoter, Ad[CD40L], was studied in prostate cancer cell lines. Cells were transduced with the vector for 2 h, washed and then incubated with or without various concentrations of FK228. After 24 h of incubation, the cells were stained for CD40L expression and analyzed by flow cytometry. FK228 treatment increased the percentage of CD40L-expressing cells as well as MFI in a dose-dependent manner (Figure 4). The most pronounced effect was observed in TRAMP-C2 cells, which are normally difficult to transduce with Ad5-based vectors. At an MOI as low as 3 FFU/cell, the percentage of CD40L-expressing cells increased from 0.3% for untreated cells to 62% for FK228-treated (3 ng/ml) cells. At MOI 0.3, FK228 treatment increased CD40L expression about 3 times in LNCaP and 2 times in PC-346C.

### HDAC inhibitors affect transgene expression driven by the prostate-specific PPT promoter negatively in prostate cancer cell lines

**Figure 5:**
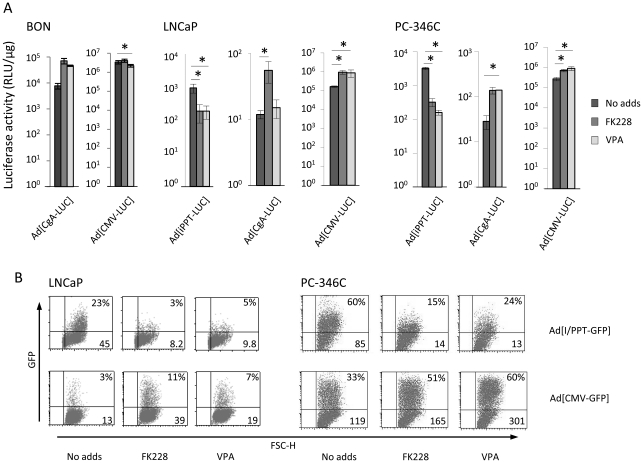
HDAC inhibitors affect tissue-specific promoters differently. **A**) BON cells were transduced with Ad[CgA-LUC] and Ad[CMV-LUC] and LNCaP and PC-346C were transduced with Ad[i/PPT-LUC], Ad[CgA-LUC] and Ad[CMV-LUC]. Cells were then incubated with 3 ng/ml FK228 or 5 mM VPA for 48 h. Both FK228 and VPA enhanced the transgene expression of the Ad[CgA-LUC] vector in BON cells about 10 times although the increase was not significant. In LNCaP and PC-346C, the expression of Ad[i/PPT-LUC] was significantly decreased while the expression of Ad[CgA-LUC] was significantly increased by HDACi treatment. **B**) LNCaP and PC-346C cells were transduced with Ad[I/PPT-GFP] and Ad[CMV-GFP] and then incubated with 3 ng/ml FK228 or 5 mM VPA for 48 h followed by flow cytometry analysis. The percentages of GFP positive cells (upper right) and MFI values (lower right) are given. GFP levels were decreased by HDAC inhibitors when the transgene expression was controlled by the PPT promoter. One representative experiment out of three is shown. Significance (p<0.05) is indicated with asterisks.

After finding out that FK228 increases transgene expression from various viral vectors, we wanted to investigate if FK228 had the same positive effect on adenoviral vectors with tissue-specific promoters as it has on an adenoviral vectors with the CMV promoter. The human chromogranin A (CgA) promoter was positively affected, although not significantly, by FK228 and VPA when the neuroendocrine pancreatic carcinoid cell line BON was transduced with Ad[CgA-LUC] (Figure 5A). A vector with the same transgene driven by the CMV promoter was used for comparison. Next, LNCaP and PC-346C cells were transduced with Ad[i/PPT-LUC and Ad[i/PPT-GFP] and thereafter incubated with FK228 or VPA for 48 h. Interestingly, the PPT-driven transgene expression of both luciferase (Figure 5A) and GFP (Figure 5B) was decreased when the cells were incubated with either of the two HDACi while, as before, CMV-driven GFP expression was increased when cells were treated with FK228 after transduction. VPA treatment increased the expression of both luciferase and GFP when the CMV promoter was used (Figure 5A and 5B). Of further interest, when the prostate cancer cell lines LNCaP and PC346-C were transduced with the neuroendocrine-selective Ad[CgA-LUC] vector and then treated with FK228 or VPA, we found that transgene expression was increased.

**Figure 6:**
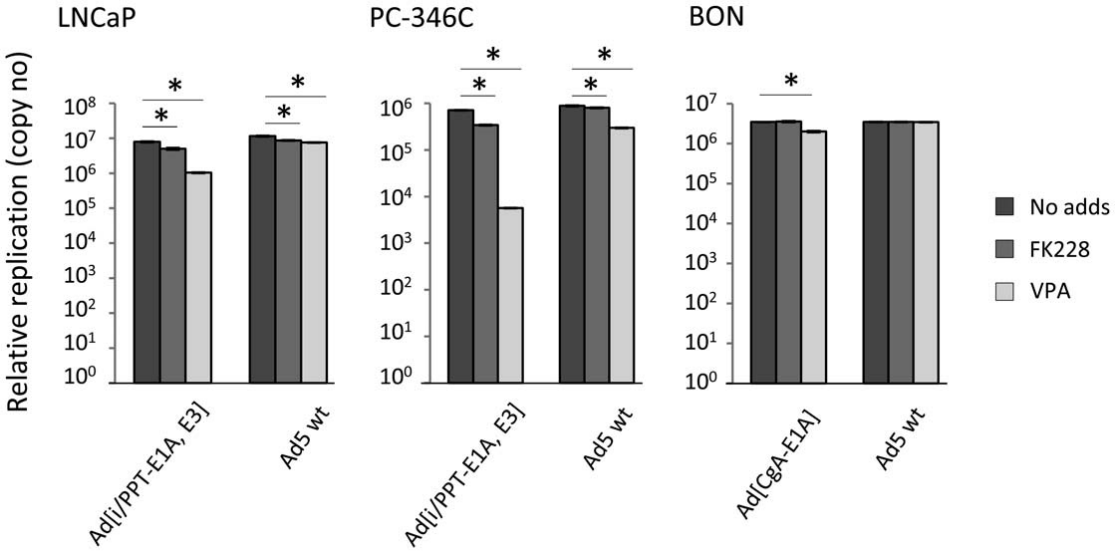
Virus replication is decreased by treatment of HDAC inhibitors. LNCaP and PC-346C were transduced with Ad[i/PPT-E1A, E3] and Ad5 wt and BON cells were transduced with Ad[CgA-E1A] and Ad5 wt. The cells were then incubated in medium with 3 ng/ml FK228 or 5 mM VPA for 72 h. Viral copy numbers were determined by quantitative PCR and related to the values obtained 2 h after transduction. Replication was decreased by FK228 and VPA treatment in most cases. Significance (p<0.0001) is indicated with asterisks.

Since reporter gene expression of PPT-based vectors was negatively affected by FK228 and VPA, we also wanted to study if replication of a PPT-controlled oncolytic adenovirus was decreased as well. LNCaP and PC-346C cells were transduced with the conditionally replicating Ad[i/PPT-E1A, E3] and Ad5 wt and BON cells were transduced with Ad[CgA-E1A] and Ad5 wt. The cells were then incubated in medium containing either FK228 or VPA for 72 h. Viral copy numbers were determined by quantitative PCR. Replication of the PPT-driven virus was decreased by FK228 in LNCaP (1.6 times) and PC-346C (1.3 times). VPA treatment reduced replication to a higher extent; 7.7 times in LNCaP and 126 times in PC-346C (Figure 6). In BON cells, a decrease (1.7 times) in replication was observed in Ad[CgA-E1A]-transduced cells treated with VPA (Figure 6). Replication of Ad5 wt was not affected in BON cells but reduced in LNCaP and PC-346C.

### HDACi differentiate virus-transduced prostate cancer cells along the neuroendocrine lineage

**Figure 7:**
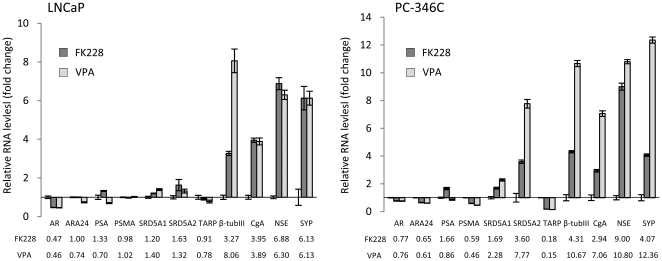
Differential gene expression in virus-transduced prostate cancer cells after 48 h of treatment with HDAC inhibitors. LNCaP and PC-346C were transduced with Ad[i/PPT-LUC] and then incubated with 3 ng/ml FK228 or 5 mM VPA for 48 h. RNA was extracted, cDNA was produced and gene expression was evaluated by quantitative PCR (triplicate samples). Gene expression in FK228 and VPA-treated cells was related to untreated cells. A two-fold difference in gene expression was set as a limit for true difference. The expression of prostate-associated genes (AR, ARA24, PSA, PSMA, SRD5A1, SRD5A2 and TARP) was not considerably affected by the HDACi. The expression of neuroendocrine-associated genes (β-tubIII, CgA, NSE and SYP) was increased in HDACi-treated cells.

The increased transgene expression from Ad[CgA-LUC] in the prostate cancer cell lines LNCaP and PC346-C and the decreased expression of Ad[i/PPT-LUC] upon HDACi treatment led us to further investigate the effect of FK228 or VPA on these cell lines. RNA expression of the genes normally controlled by the regulatory elements of PPT: PSA, PSMA and TARP were therefore evaluated along with genes involved in the androgen receptor pathway: AR, ARA24, SRD5A1 and SRD5A2, since the PPT promoter is partly regulated by testosterone. The prostate epithelial cell-associated genes: AR, ARA24, PSA, PSMA, SRD5A1, SRD5A2 and TARP were generally not affected (less than 2-fold up- or down-regulation) by the FK228 and VPA treatment. However, in LNCaP, the expression of AR was slightly decreased to just below a 2-fold down-regulation for both HDACi. In PC-346C, the expression of SRD5A1 was increased after VPA treatment and SRD5A2 expression was increased after both FK228 and VPA treatment while PSMA expression (VPA treatment) and TARP expression (FK228 and VPA treatment) were decreased (Figure 7).

We observed that the activity of the CgA promoter increased by treatment of FK228 and VPA in LNCaP and PC-346C. Furthermore, VPA is known to induce neuroendocrine differentiation in LNCaP by upregulation of β-tubIII, NSE and SYP [26], [27]. RNA levels of these elements were therefore also assessed. Therefore, expressions of the neuroendocrine-associated genes: β-tubIII, CgA, NSE and SYP were evaluated. We found them all to be greatly increased in HDACi-treated LNCaP and PC-346C cells (Figure 7), indicating that HDACi promotes differentiation along the neuroendocrine lineage for these adenocarcinoma cell lines.

## Discussion

HDACi have been shown to increase the effect of adenoviral-mediated gene therapy although the mechanisms are not fully understood [13], [14], [28]. Upregulation of CAR, the primary receptor for Ad5 infection, has been suggested as a possible mechanism [7], [15], [16], [17], [18]. However, in those studies the amount of CAR was determined by measurement of RNA levels by quantitative real-time PCR or protein levels by western blot analysis. Determination of RNA and total protein levels does not determine receptor functionality and the results can therefore be misleading. We measured instead the quantity of CAR and integrins, which are also needed for Ad5 infection on the surface of cells by flow cytometry, since it is the most appropriate technique to address receptor expression. We found that neither CAR nor integrins were upregulated after 48 h of treatment with non-toxic concentrations of FK228. Furthermore, when prostate cancer cells were transduced with Ad[CMV-GFP], the amount of GFP-expressing cells was much higher when FK228 treatment was given after transduction compared to when cells had been pre-incubated with FK228. These two observations strongly suggest that transgene expression is enhanced by a transduction-independent mechanism.

Association of cellular histones with the viral genome DNA in infected cells remains controversial [29], [30], [31], [32]. However, by using chromatin immunoprecipitation (ChIP) assays, Komatsu et al recently showed that viral chromatin is associated with both viral protein VII and cellular histones with post-translational modification (acetylation of histone H3) in infected cells [33]. They also showed that acetylation of histone H3 occurs at the promoter regions of active viral genes. Among a variety of histone modifications, acetylation at K9 and/or K14 of histone H3 is in fact recognized as an active chromatin mark for transcription [34]. It is therefore possible that FK228 affects histones associated with viral DNA and therefore enhances adenoviral transgene expression at the transcriptional level. However, this remains to be investigated.

Since we observed that FK228 administered directly after adenoviral transduction had a visible effect on transgene expression, we went on to investigate the effect of FK228 in a number of experimental models where it could be therapeutically beneficial. Monocytes are precursors of dendritic cells and as such, highly interesting as cancer vaccines after being modified to express tumor-associated antigens, often in the form of an adenoviral vector carrying the transgene coding for the tumor-associated antigen. For example, efficient adenoviral transduction of monocytes is highly warranted for *ex vivo* generation of T cells against specific antigens [35]. Such T cells could then be used for adoptive transfer to cancer patients. First, we observed that by using Ad5/f35 instead of Ad5 the amount of viral vector needed for transduction of monocytes could be lowered four times compared to Ad5 vectors (from 20 FFU/cell for Ad5 to 5 FFU/cell for Ad5/f35). We then asked ourselves if FK228 could improve transgene expression even further and we found that addition of FK228 after transduction doubled transgene expression both for Ad5 and Ad5/f35. These data imply that HDACi may increase the effect of adenoviral-based vaccination strategies, importantly also for Ad5/f35-based vectors.

The strategy to use adenoviral-mediated CD40L gene therapy to induce anti-tumor immunity has been employed in several cancer models [36] and it has recently been evaluated in a phase I/IIa clinical trial for bladder carcinoma [37]. In a previous study, we demonstrated that administration of Ad[CD40L] can suppress tumor growth and prolong survival of mice with subcutaneous, syngenic TRAMP-C2 tumors [38]. However, relatively high amount of the vector is needed to suppress tumor growth since mouse cells are semi-permissive for infection of human adenoviral vectors. We observed that addition of non-toxic concentrations of FK228 after transduction with Ad[CD40L] enhanced CD40L expression in a dose-dependent manner in prostate cancer cell lines in vitro. For TRAMP-C2 cells, which normally are hard to transduce with Ad5 [38], CD40L-expressing cells increased dramatically from 0.3% without treatment to 62% with FK228 treatment after viral transduction at MOI 3. This suggests that FK228 can be combined with Ad[CD40L] treatment and possibly with any other adenoviral vector when increased expression of a therapeutic gene is desired, i.e., lower amounts of adenoviral vectors would be required to achieve a therapeutic response.

The use of tissue-specific promoters is an attractive approach to restrict transgene expression of adenoviral vectors and replication of oncolytic adenoviruses to certain organs [39], [40]. We have previously reported on adenoviral vectors with the prostate cell-specific PPT promoter that consists of the PSA enhancer, the PSMA enhancer and the TARP promoter [21]. We have also shown that oncolytic Ad5 viruses with the PPT promoter selectively reduce cell viability of prostate cancer cell lines [20], [41] and we therefore wanted to study the putatively additive effect of adding HDAC inhibitors. However, we found that although an adenoviral vector with the CMV promoter yields higher transgene expression (GFP and luciferase) after treatment of LNCaP and PC-346C with the HDACi FK228 and VPA, the opposite was observed for the recombinant PPT promoter. One possible explanation for this is that the PPT promoter is directly affected by HDACi but a more plausible explanation is that HDACi affect gene expression and cell differentiation of LNCaP and PC-346C. Gene expression profiling experiments have revealed that HDACi including FK228 induce changes in genes coding for transcription/translation factors and factors involved in metabolism, differentiation and cell cycle [42], [43], [44]. Furthermore, VPA and other HDACi have been shown to promote neuroendocrine-like differentiation of LNCaP cells [26], [27]. We speculate that also FK228 induce neuroendocrine differentiation of LNCaP and that the activity of the PPT promoter therefore is decreased since its activity is restricted to normal and neoplastic prostate epithelial cells. In fact, four neuroendocrine-associated genes, β-tubIII, CgA, NSE and SYP, were upregulated in LNCaP and PC-346C after treatment with either FK228 or VPA. Furthermore, we also observed that CgA promoter-controlled transgene expression increased in LNCaP and PC-346C upon HDACi treatment after adenoviral vector transduction, again indicating cell differentiation along the neuroendocrine lineage.

VPA has previously been shown to hamper the adenovirus major late promoter thus affecting replication in a negative manner [45]. The PPT promoter did not lose its activity completely and we found that the replication of Ad[i/PPT-E1A, E3] in LNCaP and PC-346C was decreased by FK228, although the decrease was small, while VPA decreased the replication to a higher extent. The reason for this observation might be that there is still enough E1A produced by the PPT promoter to ensure efficient replication after FK228 treatment but not after VPA treatment. However, since the reporter gene expression driven by PPT was decreased and the replication was not improved it appears that it is not meaningful to use FK228 or VPA together with an oncolytic adenovirus controlled by this particular promoter. The transgene expression driven by another tissue-specific promoter, the neuroendocrine cell-specific chromogranin A (CgA) promoter [23] was evaluated in the endocrine pancreatic cell line BON. We found that the activity of this promoter behaved as the CMV promoter; the expression of the luciferase transgene increased after treatment of transduced cells with both FK228 and VPA. The replication of Ad[CgA-E1A] was not affected by FK228 but was reduced by VPA. We predict that most promoters will yield higher transgene expression in target tissues when FK228 or VPA are used in conjunction with adenoviral transduction. However, it is unlikely that HDACi will increase replication of viral vectors as neither of the two HDACi increased the replication of wild type Ad5 nor the tissue-restricted viruses investigated in the present study.

We conclude from the results in this study that the HDACi FK228 can be used to enhance CMV-driven and CgA-driven transgene expression of Ad5 and Ad5/35 viruses in cell lines and human monocytes. The mechanism for this enhancement seems to be transduction-independent since the highest transgene expression was observed when FK228 was administered after transduction. We therefore discourage from using FK228 as pre-treatment to adenoviral transduction and suggest evaluation of simultaneous administration of vector and FK228 or that FK228 is administered after vector treatment in pre-clinical animal models. HDACi may influence differentiation of cells and can therefore change the activity and selectivity of tissue-specific promoters, as was observed with the prostate cell-specific PPT promoter. For that reason, we propose careful investigation of HDACi effects on target cells and their interaction with viral vectors before usage. Since neither FK228 nor VPA resulted in increased replication of Ad5 wt and the tissue-restricted viruses, the use of HDACi is not likely to be beneficial for oncolytic therapy.

## Supporting Information

Figure S1 FK228 does not improve transgene expression of a lentiviral vector. Cell lines were transduced with LN[CMV-GFP] at 0.3 vp/cell and monocytes at 3 vp/cell followed by incubation for 48 h in medium containing 3 ng/ml FK228. The percentages of GFP positive cells (upper right) and MFI values (lower right) are given. For most cell types as well as monocytes, FK228 did not enhance GFP expression. In TRAMP-C2 however, the GFP expression was doubled in FK228-treated cells.
(1.10 MB TIF)

Table S1 (0.04 MB DOC)

Table S2 (0.03 MB DOC)
