# Supplementary figures and images for: The HDAC Inhibitor FK228 Enhances Adenoviral Transgene Expression by a Transduction-Independent Mechanism but Does Not Increase Adenovirus Replication
Source: PLoS One. 2011 Feb 17;6(2):e14700. doi: 10.1371/journal.pone.0014700 (PMC3040751; doi:10.1371/journal.pone.0014700)

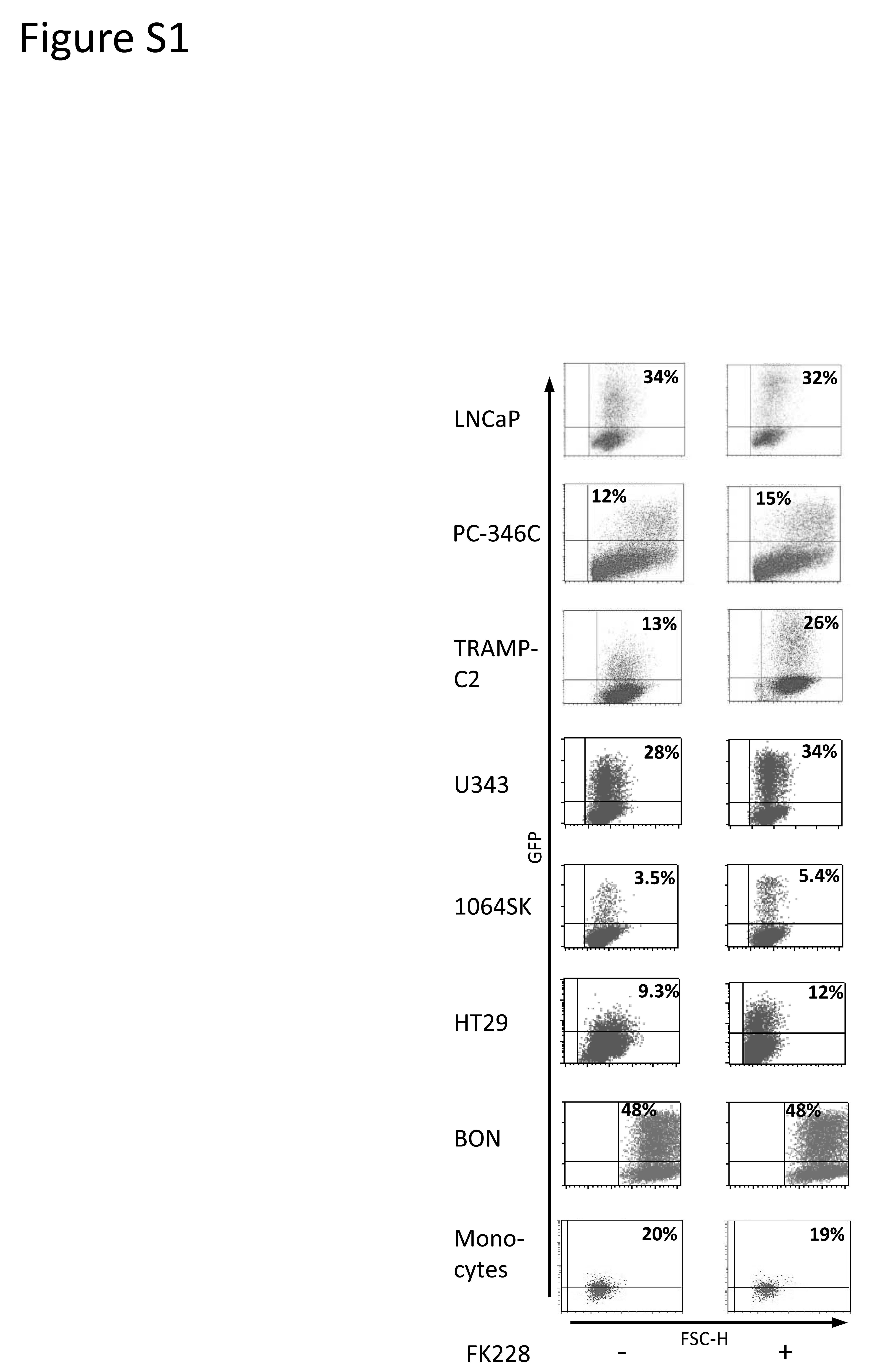

Supplement: Figure S1 — FK228 does not improve transgene expression of a lentiviral vector. Cell lines were transduced with LN[CMV-GFP] at 0.3 vp/cell and monocytes at 3 vp/cell followed by incubation for 48 h in medium containing 3 ng/ml FK228. The percentages of GFP positive cells (upper right) and MFI values (lower right) are given. For most cell types as well as monocytes, FK228 did not enhance GFP expression. In TRAMP-C2 however, the GFP expression was doubled in FK228-treated cells. (1.10 MB TIF) [file pone.0014700.s001.tif]
