# Supplementary material for: The HDAC Inhibitor FK228 Enhances Adenoviral Transgene Expression by a Transduction-Independent Mechanism but Does Not Increase Adenovirus Replication
Source: PLoS One. 2011 Feb 17;6(2):e14700. doi: 10.1371/journal.pone.0014700 (PMC3040751; doi:10.1371/journal.pone.0014700)
Supplement: Table S1 — (0.04 MB DOC) [file pone.0014700.s002.doc]

| **Table S1.** Primer sequences 5’-3’ | | | |
| --- | --- | --- | --- |
| **Symbol** | **Target gene** | **Foward primer** | **Reverse primer** |
| AR | Androgen receptor | GTGTCAAAAGCGAAATGGGC | AAACATGGTCCCTGGCAGTC |
| ARA24 | Androgen receptor coregulator | AAGTATGTAGCCACCTTGGG | AGTCCACCGAATTTCTCCTG |
| β-actin | Beta actin | CGAGAAGATGACCCAGATCATG | ACAGCCTGGATAGCAACGTACA |
| β-tubIII | Beta tubulin III | CTCTTCTCACAAGTACGTG | CCCACTCTGACCAAAGATG |
| CgA | Chromogranin A | ATACCGAGGTGATGAAATGC | AGGATCCGTTCATCTCCTC |
| NSE | Neuron-specific enolase | GCCAAAGGTCTTTTCCGG | CCTTCAGGACACCTTTGC |
| PSA | Prostate specific antigen | CCTCCTGAAGAATCGATTCC | GAGGTCCACACACTGAAGTT |
| PSMA | Prostate specific membrane antigen | ATGCCAGAGGGCGATCTAG | CTTTCCCATATCTGGCAATTAC |
| SDR5A1 | 5α reductase type 1 | AGAGCATCATGAGTGGTACC | CGCCATTGGAAAGCTTCAAG |
| SRD5A2 | 5α reductase type 2 | ACCACCATAGGTTCTACCTC | CATTGTGGGAGCTCTGCTC |
| SYP | Synaptophysin | CTACCAGCCTGACTATGG | GGGCTTCACTGACCAGAC |
| TARP | T cell receptor γ-chain alternate reading frame protein | TGCAAATGATACACTACTGC | TCTAAGCAGACAGCAGGTG |
