# Supplementary material for: The HDAC Inhibitor FK228 Enhances Adenoviral Transgene Expression by a Transduction-Independent Mechanism but Does Not Increase Adenovirus Replication
Source: PLoS One. 2011 Feb 17;6(2):e14700. doi: 10.1371/journal.pone.0014700 (PMC3040751; doi:10.1371/journal.pone.0014700)
Supplement: Table S2 — (0.03 MB DOC) [file pone.0014700.s003.doc]

| **Table S2.** Expression of surface receptors for adenoviral transduction. | | | | |
| --- | --- | --- | --- | --- |
|  | **CAR** | **αVβ3** | **αVβ5** | **CD46** |
| **LNCaP** | 0.82 | 1.19 | 0.47 | 0.82 |
| **PC-346C** | 0.97 | 1.11 | 0.75 | 0.54 |
| **1064SK** | 1.99 | 1.53 | 0.87 | 1.33 |
| **BON** | 1.03 | 0.94 | 0.76 | 0.91 |
| **U343** | 1.48 | 1.17 | 1.09 | 0.95 |
| **HT29** | 1.07 | 1.04 | 1.22 | 1.02 |
| **TRAMP-C2** | 0.95 | 0.66 | 0.81 | 0.83 |
| Mean fluorescence intensity of receptor expression of FK228 treated cells related to untreated cells (1.00). | | | | |
